# Predictive value of cervical cytokine, antimicrobial and microflora levels for pre-term birth in high-risk women

**DOI:** 10.1038/s41598-019-47756-7

**Published:** 2019-08-02

**Authors:** Rashmi Manning, Catherine P. James, Marie C. Smith, Barbara A. Innes, Elaine Stamp, Donald Peebles, Mona Bajaj-Elliott, Nigel Klein, Judith N. Bulmer, Stephen C. Robson, Gendie E. Lash

**Affiliations:** 1 Reproductive and Vascular Biology Group, Institute of Cellular Medicine, Newcastle University, Newcastle upon Tyne, NE2 4HH UK; 2 Research Department of Maternal and Fetal Medicine, Institute for Women’s Health, University College London, London, UK; 3 Infection, Immunity, Inflammation and Physiological Medicine, Institute of Child Health, University College London, London, UK; 4 Institue of Health and Society, Newcastle University, Newcastle upon Tyne, UK; 5 Guangzhou Institute of Pediatrics, Guangzhou Women and Children’s Medical Center, Guangzhou, China

**Keywords:** Intrauterine growth, Prognostic markers

## Abstract

Spontaneous preterm birth (sPTB, delivery <37 weeks gestation), accounts for approximately 10% of births worldwide; the aetiology is multifactorial with intra-amniotic infection being one contributing factor. This study aimed to determine whether asymptomatic women with a history of sPTB or cervical surgery have altered levels of inflammatory/antimicrobial mediators and/or microflora within cervical fluid at 22–24 weeks gestation. External cervical fluid was collected from women with history of previous sPTB and/or cervical surgery at 22–24 weeks gestation (n = 135). Cytokine and antimicrobial peptides were measured on a multiplex platform or by ELISA. qPCR was performed for detection of 7 potentially pathogenic bacterial species. IL-8 and IL-1β levels were lower in women who delivered preterm compared to those who delivered at term (IL-8 *P* = 0.02; IL-1β *P* = 0.04). There were no differences in elafin or human beta defensin-1 protein levels between the two groups. Multiple bacterial species were detected in a higher proportion of women who delivered preterm than in those who delivered at term (*P* = 0.005). Cervical fluid IL-8 and IL-1β and microflora have the potential to be used as biomarkers to predict sPTB in high risk women.

## Introduction

Pre-term birth (PTB), defined as delivery before 37 completed weeks of gestation, accounts for approximately 70% of neonatal deaths and is a major cause of neonatal morbidity including respiratory distress syndrome, necrotising enterocolitis and long-term neurological disabilities^1,2^. Worldwide there are 15 million preterm births every year (~1 in 10 of all births), with the annual rate increasing^3–5^. PTB may be related to spontaneous preterm labor (40–45%) or preterm prelabor rupture of membranes (25–30%), together classified as spontaneous PTB (sPTB), with the remaining preterm deliveries being indicated if either the mother or fetus is at risk^2^.

Spontaneous labor occurs following the activation of a pro-inflammatory ‘common pathway’ with increased cytokines and prostaglandins within myometrium, amniotic membranes and cervix leading to increased uterine contractility, rupture of membranes and cervical dilatation^6^. While this pathway is activated physiologically in term labor, it is postulated that one or more pathological processes (intra-amniotic infection (IAI), decidual haemorrhage, decidual senescence, disruption of maternal-fetal tolerance, decline in progesterone action, uterine over-distension and stress) may lead to activation of this pathway in preterm labor^6^. IAI is the only one which has been causally linked to sPTB; it is present in up to 30% of sPTB cases and in 60% of patients delivering before 28 weeks gestation^7,8^. When bacteria are isolated from amniotic fluid in cases of PTB, they are similar to pathogenic bacteria found in the lower genital tract suggesting the most common mode of infection is *via* the vagina through the cervix and chorioamniotic membranes^6,9–15^. Presence of a greater spectrum of bacterial species is also associated with sPTB, with 2 or more bacterial species being reported in over 60% of fetal membranes and placental tissues sampled from patients delivering preterm^15^. The cervix acts as a barrier to ascending infection and IAI is found in more than 50% of women presenting with mid-trimester cervical dilatation^16^.

Accurate prediction of sPTB allows identification of high-risk women for whom appropriate interventions such as antibiotics, cervical cerclage or progesterone can be considered, with the aim of preventing sPTB and improving neonatal outcomes. Risk factors for sPTB include a previous history of sPTB, mid-trimester pregnancy loss or cervical surgery, and strategies for prediction include screening for bacterial vaginosis and serial transvaginal ultrasound measurement of cervical length^17–20^. More recently biomarkers within cervicovaginal fluid (CVF) have been investigated as predictors of sPTB, including pro-inflammatory cytokines and natural antimicrobials such as elafin and human beta defensins (HBD), but the results have been variable with poor agreement between studies^21–33^. To date there is no established marker for predicting sPTB in asymptomatic patients. Fetal fibronectin assessment is becoming more widely used, but it is not yet recommended for use by ACOG or NICE guidelines^34,35^.

The aim of this study was to prospectively investigate whether asymptomatic women with a singleton pregnancy and a history of sPTB and/or cervical surgery had altered expression of cytokines and antimicrobial peptides, and/or microflora within cervical fluid at 22–24 weeks gestation.

## Materials and Methods

### Subjects

One hundred and thirty-five women attending PTB clinics due to either a history of previous sPTB or one or more large loop excision of the transformation zone (LLETZ) of the cervix were recruited at the Newcastle upon Tyne Hospitals National Health Service Foundation Trust (Newcastle upon Tyne, UK). The study received ethical approval (County Durham & Tees Valley 2 Research Ethics Committee; 08/H0908/79), and all patients gave informed written consent. The study was performed according to all the relevant institutional guidelines and according to the Declaration of Helsinki. Women who were under 16 years of age, had a multiple pregnancy, did not speak English or who had active vaginal bleeding were excluded from the study. Exclusions were based on the patient’s ability to give informed consent and welfare. All women were screened (and where deemed clinically appropriate treated) for lower genital tract infection at booking (high vaginal swab for microscopy, culture and sensitivity) and cervical length was measured by transvaginal ultrasound at 18–23 weeks.

### Sampling collection and processing

CVF and cells were collected from the external cervical os by gently rotating a Rovers® Cervex-Brush (Rovers Medical Devices, Lekstraat, The Netherlands) three times in the cervical canal during sterile speculum examination at 22–24 weeks gestation^36^. Following collection, cytobrush heads were cut in half with a sterile scalpel blade; one half was immediately suspended in 5 ml sterile phosphate-buffered saline (PBS) with 50 µl penicillin/streptomycin and 50µl L-glutamine, while the other half was stored in a dry pot at −20 °C. CVF was mixed well to disperse cells from the cytobrush head, centrifuged at 1800rpm for 10 minutes, supernatant collected and frozen at −80 °C.

### Sample concentration and assessment of total protein concentration

CVF was concentrated using filter concentrators with a 3 kDa cut off (Millipore, Watford, UK) according to the manufacturer’s instructions. Total protein concentration was determined by Bio-Rad Protein Assay (Bio-Rad Laboratories, Hemel Hempstead, UK).

### FAST quant multiplex arrays for cytokine analysis

FASTQuant Human II multiplex protein arrays for IL-1β, IL-2, IL-4, IL-6, IL-8, IL-10, IL-12p70, GM-CSF, MCP-1 and RANTES were run according to the manufacturer’s instructions (Whatman GE Healthcare, Sanford, ME, USA) as previously described^37,38^. The dynamic ranges for analytes were: 3–3000 pg/ml (IL-1β, IL-2, IL-6, GM-CSF), 4–3000 pg/ml (IL-4), 5–3000 pg/ml (MCP-1, RANTES), 10–3000 pg/ml (IL-8), 30–12000 pg/ml (IL-10, IL-12p70).

### ELISA for elafin and HBD1-3

ELISA was performed in duplicate to assess levels of elafin (R&D Systems, Abingdon, UK) and HBD1-3 (Peprotech, London, UK) according to the manufacturer’s instructions. The dynamic ranges were 31.2–2,000 pg/mL (elafin), 4–1,000 pg/ml (HBD1), and 16–2,000 pg/ml (HBD2, HBD3).

### DNA extraction and PCR

After thawing of the dry part of the cytobrush, 1000 µl PCR grade water (Sigma-Aldrich) was added to each container. After vortexing well, the fluid was aspirated, transferred to a sterile Eppendorf, and genomic DNA extracted using a QIAmp DNA Kit (Qiagen, Hilden, Germany) following the manufacturer’s instructions, with an additional bead-beating step to lyse bacterial cells using Lysing Matrix B and a FastPrep instrument (MP Biomedicals, Santa Ana, CA, USA) and a TissueLyser LT instrument. Samples were eluted in 200 µl UV irradiated AE (10 mM Tris-Cl, 0.5 mM EDTA). A negative control (200 µl UV irradiated buffer AE) was included in each extraction round.

Taqman PCR assays were carried out using the ABI Prism 700 detection system (Life Technologies, Paisley, UK). Each reaction consisted of QuantiTect Multiplex Mastermix with Rox Dye (Qiagen), primers and probe (final concentration of each 0.1 µM; Life Technologies; Table 1), 7 µl template DNA or control and PCR grade water to a final volume of 20 µl. Cycling conditions were: 50 °C for 2 min, 95 °C for 10 min and then 45 cycles of 95 °C for 15 sec and 60 °C for 1 min. To control for adequate DNA extraction and amplification, a real-time PCR that detects human C-reactive protein was run for each sample. Human C-reactive protein DNA was amplified in all samples.

**Table 1:** Primer/probe sets used for 16s rDNA PCR.

Primer/probe	Sequence
***Fusobacterium spp****.*	
forward	5′ GGA TTT ATT GGG CGT AAA GC 3′
reverse	5′ GGC ATT CCT ACA AAT ATC TAC GAA 3′
probe	FAM 5′CTC TAC ACT TGT AGT TCC G 3′ TAMRA
***Group B Streptococci***	
forward	5′ ATC CTG AGA CAA CAC TGA CA 3′
reverse	5′ TTG CTG GTG TTT CTA TTT TCA 3′
probe	JOE 5′ ATC AGA AGA GTC ATA CTG CCA CTT 3′ TAMRA
**Mycoplasma hominis**	
forward	5′ ATT GAT TGC TGC AGG TGA TAC A 3′
reverse	5′ GGT GTT ACA ATA TCA GCC CCA AC 3′
probe	FAM 5′ AGA GCA GCG GCA GTT GAA 3′ TAMRA
***Peptostreptococcus micros***	
forward	5′ GCC GTA AAC GATGAG TGC TAG G 3′
reverse	5′ CCA GGC GGA ATG CTT AGT GT 3′
probe	FAM 5′ TGG GAG TCA AAT CTC GCC G 3′ TAMRA
**Sneathia/Leptotricia**	
forward	5′ AAT TAT TGG GCT TAA AGG GCA TC 3′
Reverse (S)	5′ CTA CAA AAC TGT ATA ACT AGA GTA CT 3′
Reverse (L)	5′ CTA CAA AAC TGT TGA ACT AGA GTA C 3′
probe	FAM 5′ ACA AGT TGA AGG TGA AAA CCT RTG GC 3′ TAMRA
***Ureaplasma parvum***	
forward	5′CAT TGA TGT TGC ACA AGG AGA AA 3′
reverse	5′ TTA GCA CCA ACA TAA GGA GCT AAA TC 3′
probe	FAM 5′ TTG ACC ACC CTT ACG AG 3′ TAMRA
***Ureaplasma urealyticum***	
forward	5′ ATC GAC GTT GCC CAA GGG GA 3′
reverse	5′ TTA GCA CCA ACA TAA GGA GCT AAA TC 3′
probe	JOE 5′ TTG TCC GCC TTT ACG AG 3′ TAMRA
***Human C reactive protein***	
forward	5′ CTT GAC CAG CCT CTC TCA TGC 3′
Reverse	5′ TGA AGT AGA CCC CAC CC 3′
Probe	JOE 5′ TTT GGC CAG ACA GGT AAG GGG CAC 3′ TAMRA

### Placental pathology

After delivery, placentas were submitted for histopathological examination. Placentas were fixed in formalin for 48 hours, weighed and subjected to macroscopic examination for macroscopic evidence of chorioamnionitis, infarction or other lesions. Two blocks each were then taken from umbilical cord (close to the insertion and distally), reflected amniochorionic membranes and full thickness placental parenchyma to include chorionic plate and basal plate, as well as any macroscopic lesions identified. Samples were routinely processed into paraffin wax and 4 µm sections were stained with haematoxylin and eosin and examined by an experienced placental histopathologist (JNB). Placentas were examined for features of chorioamnionitis, villitis, vascular problems, placenta neoplasms such as chorangioma and miscellaneous lesions such as intervillous thrombus. Maternal and fetal inflammatory responses were staged and graded according to Redline *et al*.^39^, noting the presence or severity of chorionic vasculitis, umbilical vasculitis, subchorionitis and chorioamnionitis. Villitis was recognised by the presence of acute or chronic inflammatory cells in chorionic villous stroma. Evidence of maternal vascular underperfusion was recognised by a small placenta (<10^th^ percentile), villous infarction, increased syncytial knots, villous agglutination, distal villous hypoplasia, increased villous cytotrophoblast, thickened trophoblast basement membrane, increased intervillous fibrin and maternal vascular lesions such as acute atherosis and muscularised basal plate arteries^40,41^.

### Statistical analysis

All cytokine, elafin and HBD levels were corrected for total protein and results presented as pg/mg total protein. Statistical analyses were performed using the Prism statistical software package. Data were checked for normality and considered to be not normally distributed. Significance between two groups was determined using Mann Whitney U Test for non-parametric data. Chi-squared analysis was used to determine the association between number of bacterial species and PTB. Differences were considered significant at *P* < 0.05. A Bonferroni correction was used where multiple analyses were being performed (bacterial species and cytokine analysis; bacterial species and PTB incidence) and differences were considered significant at *P* < 0.01.

## Results

### Demographics

Of the 135 patients recruited into the study, 92 had a history of previous preterm birth alone, 34 a history of cervical surgery alone and 9 a history of both previous preterm birth and cervical surgery. Demographic details of the women recruited into the study organised by inclusion criteria and birth outcome are shown in Tables 2 and 3, respectively. Overall there were no statistically significant differences between the patient characteristics amongst the three groups of women recruited into the study.

**Table 2:** Demographics of total cohort of patients included in cervical fluid analysis.

Variable	Previous preterm birth (n = 92)	Cervical surgery (n = 34)	Both previous preterm birth and cervical surgery (n = 9)
Age (years), median (range)	29 (17–44)	33.0 (25–39)	32.0 (29–36)
BMI (kg/m^2^), median (range)	26 (17–38)	25.5 (21–52)	25 (23–27)
**Ethnicity, n (%)**			
White European	87 (94.5)	34 (100)	9 (100)
White/black Caribbean	1 (1.1)	0 (0)	0 (0)
Asian-British	1 (1.1)	0 (0)	0 (0)
Indian	2 (2.2)	0 (0)	0 (0)
Other – Philipino	1 (1.1)	0 (0)	0 (0)
**Smoking status, n (%)**			
Yes	28 (30.4)	5 (14.7)	1 (11.1)
No	64 (69.6)	28 (82.4)	8 (88.9)
Not known	0 (0)	1 (2.9)	0 (0)
Parity, median (range)	1 (0–7)	0 (0–4)	2 (1–3)
Gestational age of previous PTB (completed weeks), mean (range)	31 (23–35)	N/A	33 (27–35)
**Randomised to Opptimum trial, n (%)**			
Yes	16 (17.4)	1 (2.9)	4 (44.5)
No	76 (82.6)	33 (97.1)	5 (55.6)
**Cervical length measured, n (%)**			
Yes	54 (58.7)	15 (44.1)	5 (55.6)
No	38 (41.3)	19 (55.9)	4 (44.4)
Cervical length (mm), median (range)	31 (1–51)	30 (21–46)	36 (20–36)
**Preterm birth (this pregnancy) n (%)**			
Yes	36 (39.1)	4 (11.7)	2 (22.2)
No	56 (60.9)	30 (88.3)	7 (77.8)
Gestational age at delivery (completed weeks), median (range)	38.0 (25–42)	39.0 (28–41)	38.0 (25–41)
**Placental pathology, n (%)**			
No placental pathology	12 (13.0)	5 (14.7)	3 (33.3)
Chorioamnionitis	19 (20.7)	2 (5.9)	0 (0)
Other placental pathology	12 (13.0)	5 (14.7)	1 (11.1)
Placenta not available	49 (53.3)	22 (64.7)	5 (55.6)
**Microflora measured, n (%)**			
Yes	63 (68.4)	21 (61.8)	4 (44.4)
No	29 (31.5)	13 (38.2)	5 (55.6)

**Table 3:** Demographics of patients included in the analysis based on pregnancy outcome.

Variable	Delivery <33 weeks (n = 17)	Delivery 33–36 + 6 weeks (n = 27)	Delivery ≥37 weeks (n = 90)
Age (years), mean (range)	29 (20–39)	30.1 (22–39)	30.9 (17–44)
BMI (kg/m^2^), mean (range)	24.7 (17–37)	26.3 (19–38)	26.7 (19–52)
**Ethnicity, n (%)**			
White European	17 (100)	26 (96.3)	86 (95.5)
White/black Caribbean	0 (0)	0 (0)	1 (1.1)
Asian-British	0 (0)	0 (0)	1 (1.1)
Indian	0 (0)	1 (3.7)	1 (1.1)
Other – Philipino	0 (0)	0 (0)	1 (1.1)
**Smoking status, n (%)**			
Yes	9 (52.9)	7 (25.9)	18 (20.0)
No	8 (47.1)	21 (77.8)	71 (78.9)
Not known	0 (0)	0 (0)	1 (1.1)
Parity, mean (range)	1.8 (0–6)	1.8 (0–4)	1.4 (0–7)
Gestational age of previous PTB (completed weeks), mean (range)	29.3 (24–35)	31.3 (24–35)	31.0 (24–36)
**Previous cervical surgery, n (%)**			
Yes	5 (29.4)	3 (11.1)	36 (40.0)
No	12 (70.6)	24 (88.9)	54 (60.0)
**Randomised to Opptimum trial, n (%)**			
Yes	3 (17.6)	5 (18.5)	17 (18.9)
No	14 (82.4)	22 (81.5)	73 (81.1)
Gestational age at delivery (completed weeks), mean (range)	29.1 (25–32)	35.0 (33–36)	39.0 (37–42)

Levels of cytokines (previous PTB n = 71, cervical surgery n = 14, previous PTB/cervical surgery n = 7; total n = 95), and elafin and HBD1-3 (previous PTB n = 91, cervical surgery n = 33, previous PTB/cervical surgery n = 8; total n = 132) were determined in CVF. Cervical microflora were analysed in 88 women (previous PTB n = 63, cervical surgery n = 21, previous PTB/cervical surgery n = 4). It was not possible to analyse all the analytes in all of the samples. There was insufficient volume of CVF after concentration in 3 samples and for others, analysis of antimicrobial factors was prioritised over analysis of cytokines. For cytokine analysis all women who delivered preterm were included; samples from women who delivered at term were taken randomly from the cohort to include representative numbers of those who had previous preterm birth or cervical surgery, or both. PCR analysis was performed on a reduced sample number due to a freezer failure leading to loss of dry cytobrush samples prior to bacterial DNA analysis.

### Cervical fluid levels of cytokines, elafin and HBD1-3

Levels of IL-2, IL-4, IL-6, IL-10, IL-12p70, GM-CSF, MCP-1, RANTES, HBD2 and HBD3 in CVF were below the level of detection for the assay, despite concentration of the samples. The median (IQR; range) levels for cytokines, elafin and HBD1 are shown in Table 4. There were no differences between women who had previous sPTB or cervical surgery (data not shown).

**Table 4:** Median (IQR; range) levels for cytokines, elafin and human beta defensin 1 (pg/mg protein).

	Risk factor	Preterm delivery	Term delivery	*P* Value^a^
IL-8	Previous PTB and/or Cervical Surgery (n = 95)	991.8 (1221.3; 29.8–10963.2)	1472.4 (1710.9; 287.5–13347.2)	**0.03**
IL-1β	Previous PTB and/or Cervical Surgery (n = 95)	35.6 (78.0; 0.9–2097.9)	66.7 (190.8; 2.1–2523.1)	**0.04**
Elafin	Previous PTB and/or Cervical Surgery (n = 132)	1349.4 (2973.5; 31.6–19285.3)	792.2 (2137.1; 16.7–104485.2)	0.1
hBD1	Previous PTB and/or Cervical Surgery (n = 132)	342.8 (542.1; 24.6–23431.3)	259.6 (321.5; 17.9–7431.2)	0.2

^a^Comparison between PTB and term birth for each cytokine or antimicrobial, Mann-Whitney U test, significance at *P* < 0.05.

**Figure 1:**
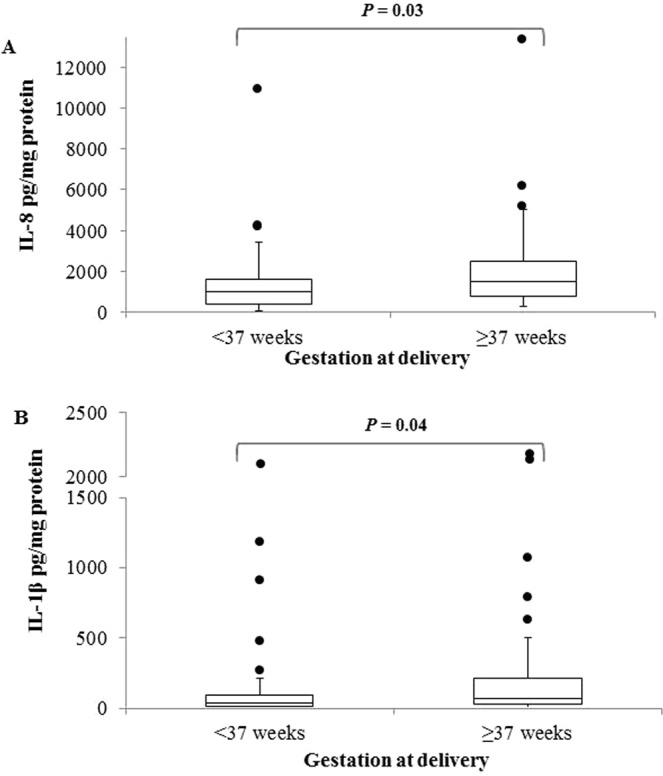
(**A**) Levels of IL-8 (pg/mg protein) in cervical fluid in women with previous PTB and/or cervical surgery (<37 weeks, n = 44; >37 weeks, n = 90). (**B**) Levels of IL-1β (pg/mg protein) in cervical fluid in women with previous PTB and/or cervical surgery (<37 weeks, n = 44; >37 weeks, n = 90).

IL-8 and IL-1β levels were reduced in women who delivered preterm (IL-8: 991.8 pg/mg protein; IL-1β: 35.6 pg/mg protein) compared with those delivering at term (IL-8: 1472.4 pg/mg protein, *P* = 0.03; IL-1β: 66.7 pg/mg protein; *P* = 0.04) (Fig. 1A,B).

Elafin and HBD1 levels did not differ between women who delivered preterm and those who delivered at term.

### Cervical microflora

**Figure 2:**
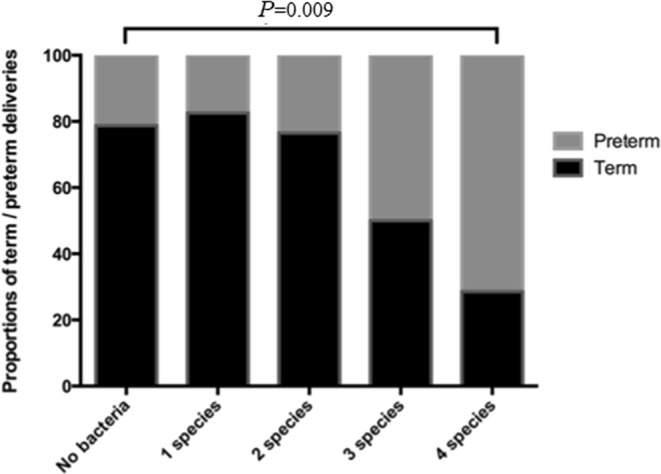
Bacterial species specific PCR for *Fusobacterium species*, *Peptostreptococcus micros*, *Group B streptococcus*, *Mycoplasma hominis*, *Ureaplasma urealyticum* and *Ureaplasma parvum* were carried out on samples collected between 22 and 24 weeks (n = 113). The proportion of women with samples positive for none of the bacteria (n = 33), one species (n = 46), two species (n = 17), three species (n = 10) and four species (n = 7) delivering preterm is represented by the grey portion of the bar. The proportion of women delivering at term is represented by the black portion of the bar. The proportion of women with samples positive for four species delivering preterm (71.4%) was significantly higher than that of the women with samples negative for all samples tested (21.42%, *P* = 0.009; Bonferroni alpha = 0.0125).

27.9% more women with samples positive for *Peptostreptococcus* (95% CI 6.0–48.6, *P* = 0.005) delivered preterm than women with samples negative for these bacteria (Table 5). There was an association between preterm birth and the number of bacterial species detected. Fifty percent more women with four or more positive samples delivered preterm compared with women with no positive samples (95% CI 4.27–78.0, *P* = 0.009; Bonferroni alpha = 0.01; Fig. 2).

**Table 5:** Spontaneous preterm birth rates in women with cervicovaginal samples positive for each bacterium species tested.

Bacterium species	Birth outcome	% (n)	Difference in proportions	95% CI of difference	*P* value^a^
Fusobacterium species	**PTB**	24.1 (7)	12.2%	−4.2–32.5	0.1
	**Term**	11.9 (10)			
Peptostreptococcus micros	**PTB**	51.7 (15)	27.9%	6.0–48.6	**0.005**
	**Term**	23.8 (20)			
Group B streptococcus	**PTB**	27.5 (8)	18.0%	0.97–38.4	0.02
	**Term**	9.5 (8)			
Mycoplasma hominis	**PTB**	17.2 (5)	7.7%	−6.4–27.0	0.3
	**Term**	9.5 (8)			
Ureaplasma urealyticum	**PTB**	27.5 (8)	−9.4%	−12.8–28.0	0.4
	**Term**	36.9 (31)			
Ureaplasma parvum	**PTB**	27.5 (8)	15.6%	−1.7–36.2	0.05
	**Term**	11.9 (10)			

PTB = preterm birth; CI = confidence interval.

^a^Comparison between PTB and term birth for each bacterial species (proportion of positive samples), Chi-Squared test, significance at *P* < 0.05.

### Cervical microflora and cytokine, elafin and HBD1 levels

Paired bacterial DNA samples and CVF were available for 45, 51, 81 or 87 women depending on analysis variables (Table 6). In women who tested positive for *Fusobacterium* species median levels of IL-8 (*P* = 0.01) and HBD1 (*P* = 0.007) were higher than in those women who tested negative. In women who tested positive for *Peptostreptococcus micros* the median concentration of HBD1 (*P* = 0.0005) was higher than in women who tested negative. Median levels of HBD1 were also higher in women who tested positive for *Group B streptococcus* (*P* = 0.0005) and *Ureaplasma parvum* (*P* = 0.01) than women who tested negative. No other differences were observed (Table 6).

**Table 6:** Cytokine and antimicrobial levels (Median, range) in bacterium species positive and negative samples.

Bacterium species	Bacterium status	IL-8	IL-1β	Elafin	HBD1
Fusobacterium species	**Positive**	2093.5 (360.0–13347.2; n = 17) ***P*** = **0.01**^a^	37.7 (2.5–2523.1; n = 17)	1165.7 (36.1–104485.2; n = 17)	442.6 (41.0–7431.2; n = 17) ***P*** = **0.007**
	**Negative**	678.8 (32.2–4187.8; n = 34)	32.6 (0.9–482.2; n = 34)	870.8 (16.7–19285.3; n = 70)	241.8 (17.9–2376.2; n = 70)
Peptostreptococcus micros	**Positive**	1162.9 (50.3–13347.2; n = 21)	49.2 (2.5–2523.1; n = 21	922.8 (26.6–104485.2; n = 31)	442.6 (24.6–7431.2; n = 31) ***P*** = **0.0005**
	**Negative**	638.1 (32.3–4187.8; n = 30)	17.0 (0.9–482.2; n = 30)	902.6 (16.7–19285.3; n = 56)	214.6 (17.9–2376.2; n = 56)
Group B streptococcus	**Positive**	1463.5 (331.2–2673.3; n = 14)	40.9 (4.0–482.2; n = 14)	854.6 (27.7–4491.7; n = 16)	508.9 (149.7–3382.4; n = 16) ***P*** = **0.0005**
	**Negative**	648.1 (32.3–13347.2; n = 37)	34.9 (0.9–2523.1; n = 37)	903.3 (16.7–104485.2; n = 71)	237.8 (17.9–7431.2; n = 71)
Mycoplasma hominis	**Positive**	493.2 (50.3–3012.3; n = 7)	50.5 (2.5–340.2; n = 7)	771.9 (95.31–4431.3; n = 10)	345.6 (24.6–3382.4; n = 10)
	**Negative**	1104.3 (32.3–13347.2; n = 44)	33.3 (0.9–2523.1; n = 44)	903.3 (16.7–104485.2; n = 77)	262.8 (17.9–7431.2; n = 77)
Ureaplasma urealyticum	**Positive**	370.8 (32.2–2111.7; n = 9)	82.0 (0.9–482.2; n = 9)	621.0 (16.7–7255.9; n = 27)	199.6 (24.6–1009.2; n = 27)
	**Negative**	1265.7 (62.0–3507.4; n = 36)	28.5 (2.1–2523.1; n = 36)	1077.7 (26.6–13799.5; n = 54)	302.4 (17.9–2376.2; n = 54)
Ureaplasma parvum	**Positive**	1591.7 (360.0–3507.4; n = 15) ***P*** = **0.002**	50.5 (2.5–2173.9; n = 15)	922.8 (26.6–7255.9; n = 17)	422.8 (41.0–1098.8; n = 17) ***P*** = **0.01**
	**Negative**	536.9 (32.3–3029.7; n = 30)	28.4 (0.9–2523.1; n = 30)	784.1 (16.7–13799.5; n = 64)	214.6 (17.9–2376.2; n = 64)

^a^Comparison of cytokine or antimicrobial levels in women positive or negative for each bacterial species, Mann-Whitney U test, significance at *P* < 0.01.

### Placental pathology

Placentas from 28 (66.4%) preterm and 35 (38.1%) term deliveries were available for histological examination (Table 1). A higher proportion of placental samples from women who delivered preterm showed signs of both chorioamnionitis and other placental pathology (*P* = 0.0001, Fisher’s exact test). In the women who delivered <37 weeks 20% (5/25) showed no placental pathology, 44% (11/25) had chorioamnionitis and 36% (9/25) other placental pathologies. In the women who delivered at term, 43% (15/35) showed no placental pathology, 28.5% (10/35) had some degree of chorioamnionitis and 28.5% (10/35) other placental pathologies.

**Figure 3:**
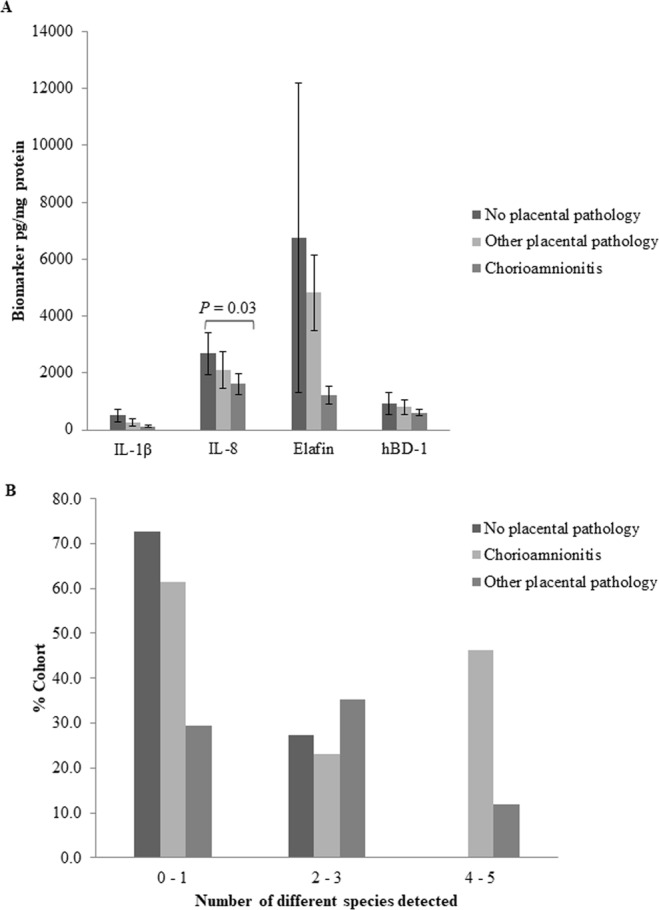
Association between placental pathology and (**A**) biomarker levels (IL-1β, IL-8, Elafin, hBD-1); and (**B**) 0–1, 2–3 or 4–5 different bacterial species present in cervical fluid.

Irrespective of pregnancy outcome, IL-8 levels were lower in the chorioamnionitis group compared to those without placental pathology (*P* = 0.03; Fig. 3A). There was no association between the presence of any species of bacterium and placental pathology (data not shown) or between the number of different detectable species and placental pathology (Fig. 3B).

## Discussion

This study demonstrated a reduction in the level of IL-8 and IL-1 β in CVF taken at 22–24 weeks gestation from high-risk asymptomatic women who subsequently delivered preterm compared with women who delivered at term. In addition, women who delivered preterm had a greater bacterial load than those who delivered at term.

Our finding of reduced IL-8 and IL-1β supports several previous studies but is in disagreement with others^32,42–46^. Cervical IL-8 and IL-1β (8–20 weeks gestation) were lowered in women who subsequently developed clinical chorioamnionits^42^. Women with low IL-1β and/or IL-8 and potentially pathological vaginal microflora had an increased risk of preterm birth, although reduced cytokines alone were not associated with PTB^43^. In addition, women with a high anti-inflammatory/low pro-inflammatory stratum in the first trimester had an increased risk of delivering before 34 weeks gestation^43^. In contrast, no change in CVF levels of IL-8 or IL-1β were reported prior to cervical shortening in women at high risk of sPTB^45^ and in another study a higher percentage of women with increased cervical mucus IL-8 levels at 20–24 weeks gestation delivered preterm compared with those with normal IL-8 levels^46^. Elevated antenatal vaginal levels of IL-8 and IL-1β in high risk women who delivered preterm compared to those delivering at term have also been reported, with IL-8 being an independent predictor of sPTB^32^. Reduced levels of cytokines within the lower genital tract in early pregnancy could indicate a broad immune hyporesponsiveness and an attenuated ability to mount an ‘appropriately vigorous’ response to infection^35^. Thus reduced maternal cervical immunity could lead to an environment more conducive to the ascent of pathogens into the choriodecidua, increasing susceptibility to chorioamnionitis^35,43^. Indeed, we also found an association between reduced cervical IL-8 levels and chorioamnionitis.

Cervical elafin levels have also not been found to be consistently altered in high risk women who deliver preterm^47–49^. One study found no correlation between CVF levels of elafin at 20–23 + 6 weeks gestation and subsequent sPTB, which is in agreement with the findings of the present study^47^. In contrast, reduced CVF elafin levels have been reported in women with bacterial vaginosis at less than 20 weeks gestation, while another study reported increased second trimester CVF elafin levels in high risk women with a short cervix and subsequent sPTB^48,49^.

The different findings in cytokine and elafin levels compared with some (but not all) other studies may be explained by differences in the gestational ages of sample collection, sampling methodology, measurement methodology, indication for study inclusion, and outcome measure. They may also reflect different genetic polymorphisms that have been reported for IL-1β and elafin^48,50–53^.

Previous work investigating human beta defensins and sPTB has focused on amniotic fluid levels, with second trimester HBD2 levels reported to be associated with preterm premature rupture of membranes, but not with preterm labor^54^. In this study cervical HBD1 levels were not altered in women delivering preterm compared with those delivering at term, and cervical HBD2 and HBD3 were not detected.

Women with cervicovaginal samples positive for *Peptostreptococcus micros* or *Group B streptococcus* and women with increasing numbers of potentially pathogenic bacterial species were more likely to deliver preterm. These species are frequently detected in membrane and placental samples from women delivering preterm, and a similar association has been demonstrated between increasing number of bacterial species detected in fetal membranes and preterm birth^15^. While *Group B streptococcus* and *Ureaplasma parvum* are not associated with BV, *Peptostreptococcus micros, Mycoplasma hominis, Fusobacterium species* and *Ureaplasma urealyticum* have all been described as BV associated bacteria^55^. Recent studies suggest an association between increased diversity of bacterial species in the vagina and PTB and the induction of the pro-inflammatory cytokines IL-1β, IL-6, IL-8, and TNF-α, which are known mediators of cervical remodeling^56,57^. In the current cohort we report increased IL-8, IL-1β and HBD1 levels in women positive for *Fusobacterium species*, *Peptostreptococcus micros, Group B streptococcus* and *Ureaplasma parvum*, and reduced levels of IL-8 and HBD1 in women positive for *Ureaplasma urealyticum*, but due to relatively low sample numbers pregnancy outcome was not taken into consideration in this analysis. In the current study we report a reduction in levels of IL-1β and IL-8 in women who deliver preterm. We also demonstrate an association between microflora load and pre-term birth, with those women positive for 4 or more different bacterium species being more likely to deliver pre-term than those with no positive sample. However, there was no association between cytokine, or antimicrobial, levels and number of positive species (data not shown). The explanation for this dichotomy in results is unclear and a much larger cohort would be needed to tease out the relative importance of these different factors.

In this cohort of high risk women there was a high level of placental pathology irrespective of gestation at delivery, although the rate of placental pathology was higher in the group that delivered preterm compared with those who delivered at term. These data may reflect a susceptibility of these women to placental infection and damage. Inflammation was staged and graded according to Redline criteria^39^ and some women had early stage maternal and/or fetal inflammation such as subchorionitis or chorioinic vasculitis. In addition, lowered cervical fluid IL-8 levels were associated with chorioamnionitis irrespective of gestation at delivery, suggesting that lowered cervical antimicrobial defenses are associated with ascending infection and placental pathology, even if these features do not always lead to preterm birth. We might therefore postulate that ascending infection and placental pathology alone are not enough to cause preterm birth and that the uterine response and/or severity of infection/placental pathology are more important indicators of whether a woman will deliver preterm or not.

A strength of this study is that the CVF was collected at an early gestational age (20–22 weeks), and therefore marker levels were not influenced by confounding factors such as uterine contractions during labor. This time was chosen as it fits with routine care visits within the UK and does not require women to attend the hospital for a non-scheduled visit. We utilised multiplex arrays when measuring cytokine levels, allowing for the concurrent measurement of numerous cytokines from the small CVF samples obtained. The major limitation of our study was that it was under powered to allow the outcome of earlier pre-term delivery (<33 weeks gestation) to be considered separately. In addition, not all samples were available for analysis in all of the different assays; due to freezer failure (microflora analysis), and limited sample volume and multiplex kit size (cytokine analysis). Biomarker levels varied substantially with large overlaps between outcome groups and hence our study was too small to produce prediction statistics, a larger replication cohort is required. The microflora study was exploratory and needs to also be repeated with a larger cohort and non-biased analytical methodology. A small number of women in the study were also included in a randomised controlled trial of vaginal progesterone (OPPTIMUM: ISRCTN14568373)^58^ and it is possible that, in those taking active drug, this impacted on outcome, although no effect of progesterone on outcome was reported^59^.

In conclusion, we have demonstrated that patients with a history of prior sPTB and/or prior cervical surgery who deliver preterm, have reduced second trimester CVF IL-8 and IL-1β levels prior to onset of sPTB symptoms. Women with four or more bacterial species in the cervix were more likely to deliver preterm. Further studies are required to understand the complex relationship between host antimicrobial protein expression and the mucosal cervical microflora. In addition, much larger studies are needed to assess whether these biomarkers independently or collectively improve prediction of sPTB in women with a history of sPTB, especially given the large variation in CVF cytokine levels which does not allow for predictive cut-off levels to be determined.
